# Aminoquinoline surfen inhibits pseudorabies virus attachment by preventing the binding of glycoprotein C to heparan sulfate

**DOI:** 10.1128/spectrum.03223-25

**Published:** 2026-03-05

**Authors:** Yiping Wang, Binbin Zhu, Xinyi Huang, Fei Zhao, Zhiyuan Zheng, Senhong Zhao, Xiaobo Huang, Yi Zheng, Qin Zhao, Yiping Wen, Rui Wu, Senyan Du, Chun Wu, Tongqing An, Sanjie Cao

**Affiliations:** 1 Research Center for Swine Diseases, College of Veterinary Medicine, Sichuan Agricultural University 506176 , Chengdu, China; 2 Agricultural Animal Diseases and Veterinary Public Health Key Laboratory of Sichuan Province, Sichuan Agricultural University 506176 , Chengdu, China; 3 Key Laboratory of Agricultural Bioinformatics of Ministry of Education, Sichuan Agricultural University 506176 , Chengdu, China; 4 Engineering Research Center of Southwest Animal Disease Prevention and Control Technology of Ministry of Education, Sichuan Agricultural University 506176 , Chengdu, China; 5 State Key Laboratory of Animal Disease Control and Prevention, Harbin Veterinary Research Institute, Chinese Academy of Agricultural Sciences 111613 , Harbin, China; 6 College of Science, Sichuan Agricultural University, Ya’an, China; University of Florida College of Dentistry, Gainesville, Florida, USA

**Keywords:** pseudorabies virus, virus attachment, glycoprotein C, surfen, heparan sulfate

## Abstract

Pseudorabies virus (PRV) is an important herpesvirus that infects pigs and many other animals. PRV can also infect humans, but the relevant reports are still very rare. PRV is not only a serious concern in veterinary medicine, but also a potential threat to public health. Thus, the development of effective antiviral agents against PRV is needed for its prevention and control. PRV utilizes viral glycoprotein C (gC) to bind heparan sulfate (HS) on target cells to complete virus attachment. Here, we identified aminoquinoline surfen, a small-molecule antagonist of HS, as a novel and potent antiviral agent against PRV. We demonstrated that surfen not only effectively inhibited PRV infection in multiple permissive pig, human, and mouse cell lines, but also significantly elevated mice survival after lethal PRV challenge. Surfen pre-treatment significantly reduced PRV attachment and infectious virus production in target cells. Enzymatic removal of HS chains by heparinase or addition of exogenous HS counteracted PRV inhibition by surfen, demonstrating that its antiviral activity is dependent on HS. Notably, surfen lost its ability to inhibit the attachment of PRV gC-deficient virus, which enters cells in an HS-independent manner, indicating that the binding of surfen to HS interferes with the interaction between PRV gC and HS on the cell surface. Cumulatively, these findings demonstrate that surfen inhibits PRV attachment by preventing the binding of PRV gC to HS on target cells and reveal that targeted disruption of PRV gC-HS interaction is an effective strategy to develop antiviral drugs to defend PRV infection.

**IMPORTANCE:**

Pseudorabies virus (PRV) causes severe respiratory, reproductive, and neurological disorders in pigs, and its infection in humans is also reported occasionally. PRV is not only a serious concern in veterinary medicine, but also a potential threat to public health. Therefore, the development of effective antiviral agents against PRV is needed to prevent its infection and spread. Here, we demonstrated that aminoquinoline surfen, a small-molecule antagonist of HS, effectively inhibited PRV infection in multiple permissive cell lines *in vitro* and significantly elevated mice survival after lethal PRV challenge *in vivo*, revealing it as a novel and potent antiviral agent against PRV. Mechanistic studies demonstrated that surfen inhibited PRV attachment by preventing the binding of PRV viral glycoprotein gC to heparan sulfate on target cells. Cumulatively, these findings reveal that targeted disruption of PRV gC-HS interaction is an effective strategy to develop antiviral drugs to defend PRV infection.

## INTRODUCTION

Pseudorabies virus (PRV), also known as suid herpesvirus 1 or Aujeszky’s disease virus, is an important member in the genus *Varicellovirus* in the subfamily *Alphaherpesvirinae* of the family *Herpesviridae* (1, 2). It is a large double-stranded DNA virus, with a genome length of approximately 143 kb, which has the capability to encode more than 70 viral proteins (3). Of the PRV-encoded proteins, viral glycoprotein B (gB), gC, gD, gH, and gL synergistically cooperate to accomplish virus attachment, fusion, and entry (4, 5). PRV gC is the major viral glycoprotein responsible for binding to heparan sulfate (HS) on the target cells (6–10). PRV gB, gD, gH, and gL mainly mediate the fusion between virion envelope and the plasma membrane (4, 5, 11, 12). PRV gD binds to one of the many cellular receptors, such as nectin 1 (13, 14), which transmits signals to the gH/gL heterodimer to activate the fusion protein gB, thus facilitating membrane fusion and viral entry into target cells (4, 5, 11, 12). It is worth noting that PRV can also enter target cells in a gC-independent manner, as demonstrated by the findings that the PRV gC-deficient virus could still enter cells in spite of having a lower efficiency compared to wild-type virus (6, 7). Attachment of the PRV gC-deficient virus is independent of HS on the cell surface, and PRV gD is mainly responsible for binding of the gC-deficient virions to cell surface receptors apart from HS, which promotes viral fusion and entry (6, 7).

PRV has a wide range of host tropism. Although pigs are identified as the natural reservoirs, PRV has also been demonstrated to infect a wide variety of livestock and wild animals, including cattle, sheep, goats, horses, chickens, raccoons, possums, skunks, rabbits, deer, dogs, cats, ferrets, wolves, guinea pigs, and rodents (3, 15). PRV infection causes severe diseases in pigs, mainly characterized by fatal encephalitis in newborn piglets and severe reproductive failure in pregnant sows, which results in enormous economic losses in the pig industry worldwide (3, 15). PRV infection of animals other than its natural swine host typically results in severe neurological signs and death (3, 16). Since 1914, there have been a few reports claiming that PRV infection in laboratory personnel could cause fever, sweating, weakness, fatigue, sore throat, itching, and central nervous system symptoms, such as difficulty in swallowing, abnormal sensations, and tinnitus (17, 18). More recently, several research groups in China reported that PRV infection among workers in pig farms caused severe endophthalmitis and encephalitis, and the presence of PRV could be detected by PCR and DNA sequencing (19–25). In 2021, a research team in China isolated the first human PRV strain, designated the hSD-1/2019 strain, from the cerebrospinal fluid of a PRV-infected patient (26). These findings clearly demonstrate that PRV has the ability to infect humans, with a potential risk of cross-species transmission from infected animals to humans, posing a potential threat to public health (27, 28). Therefore, PRV not only seriously affects the healthy and sustainable development of the global pig farming industry, but also might become a potential public health concern. To date, no drugs have been approved to defend PRV infection, and the development of antiviral agents against PRV is needed.

Surfen (bis-2-methyl-4-amino-quinolyl-6-carbamide) is a dimeric aminoquinoline that was originally developed as an excipient for the production of depot insulin (29). An early study reported that surfen had the ability to neutralize the activity of heparin in oral feeding experiments in rats (30). A subsequent screen of the National Cancer Institute Small Molecule Diversity Set identified surfen as a potent antagonist of HS and heparin (31). Surfen potently binds to HS, heparin, dermatan sulfate, and chondroitin sulfate (31). Binding of surfen to heparin neutralized the capability of heparin to activate antithrombin and blocked the sulfation and degradation of chains of glycosaminoglycan by bacterial lyases (31). Furthermore, surfen altered HS-mediated cellular processes, including growth factor binding and activation, cell attachment, and angiogenesis (31). Interestingly, surfen inhibited HS-dependent infection by herpes simplex type 1 (HSV-1) and the enhancement of human immunodeficiency virus 1 (HIV-1) infection by semen-derived amyloid fibrils (31, 32). Because PRV entry into target cells depends on HS, we hypothesized that surfen might also exert an inhibitory effect on PRV infection. However, to date, the role of surfen in PRV infection has not been determined.

In the work described here, we systematically investigate the antiviral activity of surfen against PRV infection. We demonstrated surfen as a novel and potent antiviral agent against PRV infection *in vitro* and *in vivo*. We show that surfen efficiently inhibited PRV infection in multiple permissive pig, human, and mouse cells, as well as in mice. Inhibition of PRV infection by surfen occurred in the stage of viral attachment, which was dependent on cell surface HS. Furthermore, we demonstrated that surfen failed to inhibit the attachment and production of infectious virions upon infection with PRV gC-deficient virus, which absorbs to target cells in an HS-independent fashion. These results suggest that surfen inhibited virus attachment by preventing the binding of PRV gC to HS on target cells. Cumulatively, these findings reveal surfen as a novel antiviral drug against PRV infection, which exerts antiviral activity by preventing the binding of PRV gC to HS on target cells.

## RESULTS

### Identification of surfen as a novel antiviral agent against PRV infection

**Fig 1:**
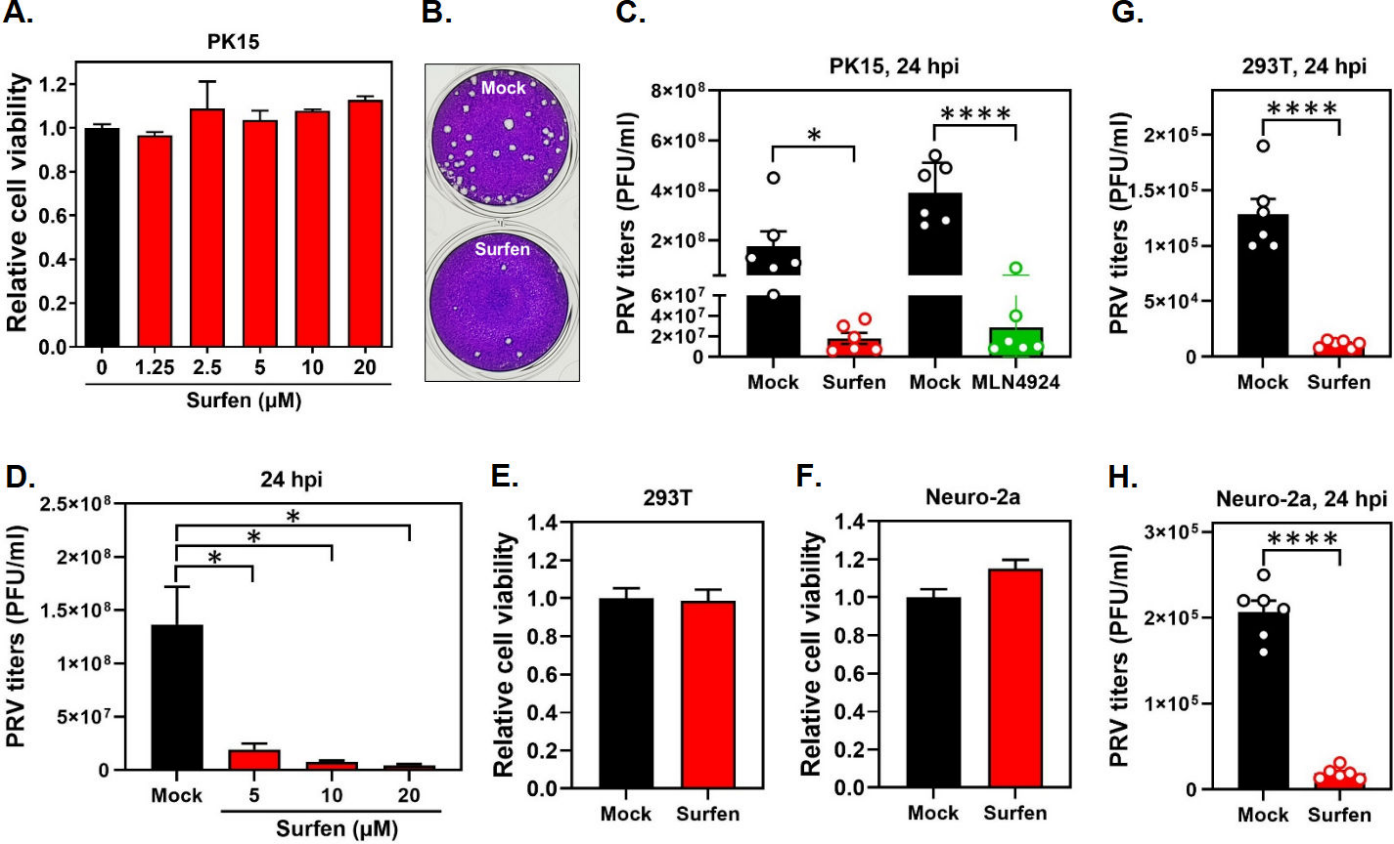
Surfen inhibits PRV infection in multiple permissive cells. (**A**) Viability of PK15 cells in the presence of surfen. PK15 cells were treated with surfen at 0, 1.25, 2.5, 5, 10, and 20 µM for 24 h, and the cell viability was determined using a WST-8-based Cell Counting Kit-8, according to the manufacturer’s instructions. (**B and C**) PK15 cells were mock treated or treated with surfen at 10 µM for 2 h, infected with PRV in the presence of surfen for 1 h, and then incubated in the continued presence of surfen for 24 h. The cells and supernatants were harvested, and viral titers were measured by plaque assay; representative plaque images are shown in panel **B**. In parallel experiments, PK15 cells were infected with PRV and then mock treated or treated with MLN4924 at 10 µM for 24 h. PRV titers were summarized in panel **C**. (**D**) The antiviral experiments were performed as described in panel **C**, except that surfen was added at different concentrations (5, 10, and 20 µM). (**E and F**) Viability of 293T (**E**) and Neuro-2a (**F**) cells in the presence of surfen at 10 µM was detected following the procedures as described in panel **A**. (**G and H**) The antiviral activity of surfen against PRV infection in 293T (**G**) and Neuro-2a (**H**) cells was determined following the procedures described in panel **C**. Values represent the means ± the standard errors of the mean (SEM) of two independent experiments. Significance was determined by a two-tailed, unpaired *t*-test (*P < 0.05, ****P < 0.0001).

To determine whether surfen would exert an antiviral activity against PRV infection, we first tested whether surfen inhibited PRV infection in PK15 cells, a permissive porcine cell line widely used for PRV pathogenesis investigation. We treated PK15 cells with different concentrations of surfen (1.25 to 20 µM) and measured cell viability by CCK-8 assay. We observed that surfen did not trigger any cytotoxicity under the concentrations tested (Fig. 1A). Subsequently, PK15 cells were treated with surfen, infected with PRV in the presence of surfen, and incubated in the continued presence of surfen for 24 h, and then, viral titers were determined by a standard plaque assay (Fig. 1B). To ensure the accuracy of our antiviral assay, the neddylation inhibitor MLN4924, which was reported to effectively inhibit PRV infection (33), was included as a positive control in parallel experiments. In agreement with the reported findings (33), we also demonstrated that MLN4924 significantly inhibited PRV replication (Fig. 1C). Interestingly, we found that PRV titers produced in surfen-treated PK15 cells were strikingly reduced compared to those of the mock group (Fig. 1C), suggesting that surfen effectively inhibited PRV infection. Importantly, the antiviral activity of surfen is comparable to that of MLN4924 (Fig. 1C). To determine whether this inhibitory effect is dose-dependent, the antiviral experiments were carried out in the presence of different concentrations of surfen (5, 10, and 20 µM). As expected, surfen inhibited PRV infection in a dose-dependent manner (Fig. 1D). Taken together, these findings demonstrate that surfen effectively inhibits PRV infection in PK15 cells.

Because PRV has a variety of host tropisms, we next sought to determine whether surfen repressed PRV infection in non-natural host cells. We utilized two other permissive cells, a human embryonic kidney cell line (293T) and a mouse neuroblastoma cell line (Neuro-2a). We first confirmed that surfen did not induce any cytotoxicity in 293T (Fig. 1E) and Neuro-2a (Fig. 1F) cells under the concentration tested. Then, we detected the antiviral effect of surfen against PRV infection in these two cell lines at 24 h post-infection (hpi). Consistent with a significant decrease in PRV titers observed in PK15 cells, surfen treatment also resulted in a marked reduction in PRV titers in 293T (Fig. 1G) and Neuro-2a (Fig. 1H) cells. Cumulatively, these findings demonstrate that surfen inhibits PRV infection in multiple permissive cells, revealing it as a novel and potent antiviral agent against PRV infection.

### Surfen inhibits PRV gene expression and DNA synthesis

**Fig 2:**
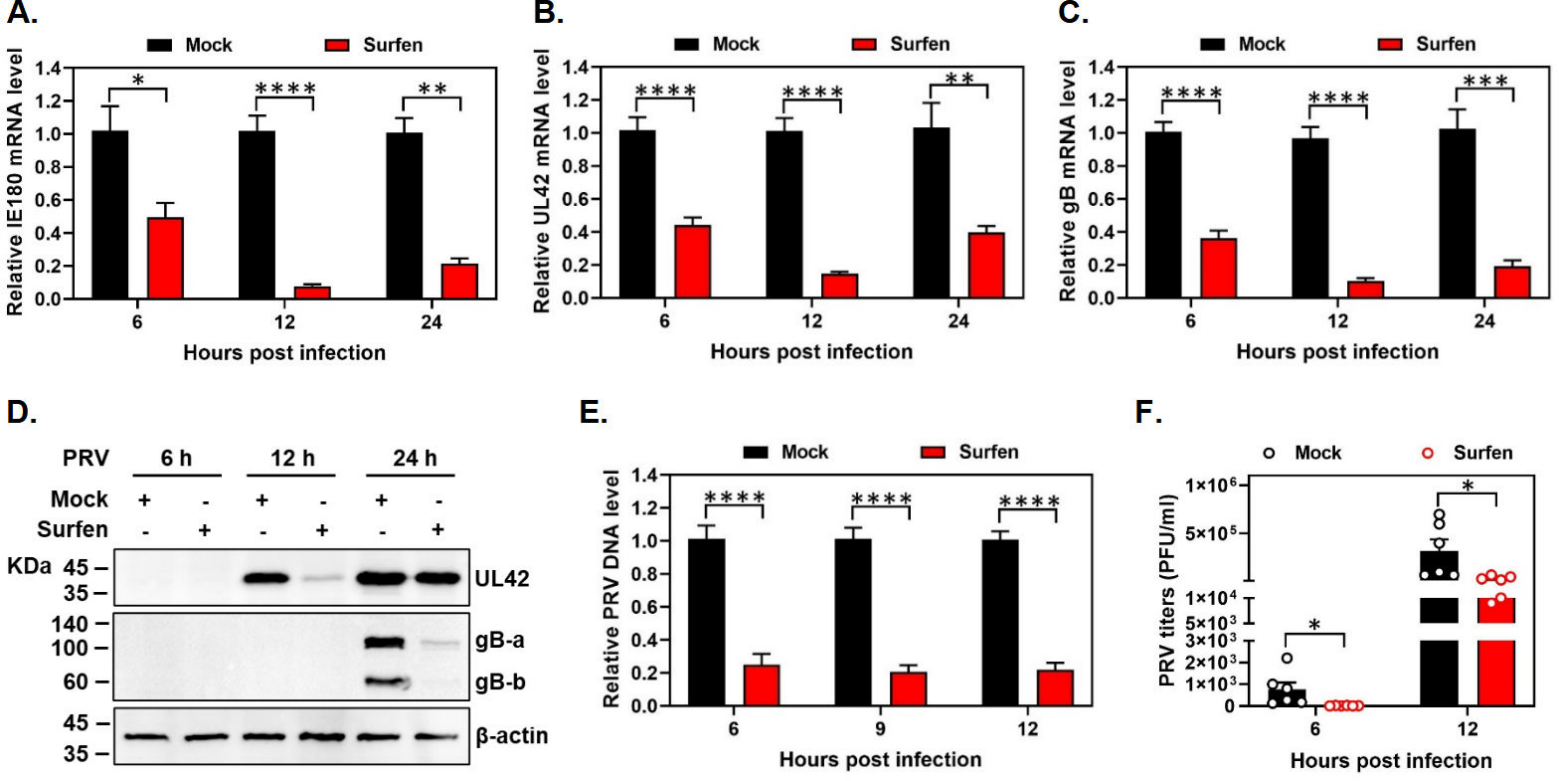
Surfen inhibits PRV gene expression and DNA synthesis. PK15 cells were mock treated or treated with surfen at 10 µM for 2 h, infected with PRV in the presence of surfen for 1 h, and then incubated in the continued presence of surfen. (**A–C**) Total RNA was extracted from the cells at 6, 12, and 24 hpi and reversed-transcribed. Then, the mRNA expression levels of viral genes *IE180* (**A**), *UL42* (**B**), and *gB* (**C**) were measured by qPCR. (**D**) Protein lysates were harvested from the cells at 6, 12, and 24 hpi. Then, the expression levels of UL42 and gB proteins were determined by Western blots. β-Actin was included as a loading control. (**E**) DNA was extracted from the cells at 6, 9, and 12 hpi. Then, the viral gB DNA copies were measured by qPCR to assess the relative PRV genomic DNA level. (**F**) The cells and supernatants were harvested at 6 and 12 hpi. Then, viral titers were determined by plaque assay. Values represent the means ± SEM of two independent experiments. Significance was determined by a two-tailed, unpaired *t*-test (*P < 0.05, **P < 0.01, ***P < 0.001, ****P < 0.0001).

Having demonstrated an inhibitory effect of surfen on the production of PRV infectious virus at 24 hpi, we next sought to systemically detect the impact of surfen on PRV gene expression and DNA synthesis at different time points. PK15 cells were treated with surfen, infected with PRV in the presence of surfen, and incubated in the continued presence of surfen, and then, we quantified the mRNA expression levels of PRV intermediate-early gene *IE180*, early gene *UL42*, and late gene *gB* by RT-qPCR at 6, 12, and 24 hpi, respectively. As compared to mock-treated cells, we observed that surfen treatment led to a significant decrease in the mRNA expression of viral genes *IE180* (Fig. 2A), *UL42* (Fig. 2B), and *gB* (Fig. 2C) at all time points tested. Consistent with a reduction in mRNA levels, surfen treatment also resulted in a significant decrease in the expression of UL42 and gB proteins (Fig. 2D). Subsequently, we assessed whether the reduction in viral gene expression by surfen resulted in a block in PRV DNA synthesis. As expected, we observed a striking decrease in the synthesis of PRV DNA in the presence of surfen at 6, 9, and 12 hpi (Fig. 2E). Moreover, the defects in PRV gene expression and DNA synthesis after surfen treatment were accompanied by a significant reduction in the production of infectious viruses at both 6 and 12 hpi (Fig. 2F). Taken together, these findings demonstrate that surfen effectively inhibits PRV gene expression and DNA synthesis and imply that surfen might exert an antiviral activity at the early stage.

### Pre-treatment of surfen inhibits PRV infection *in vitro* and *in vivo*

**Fig 3:**
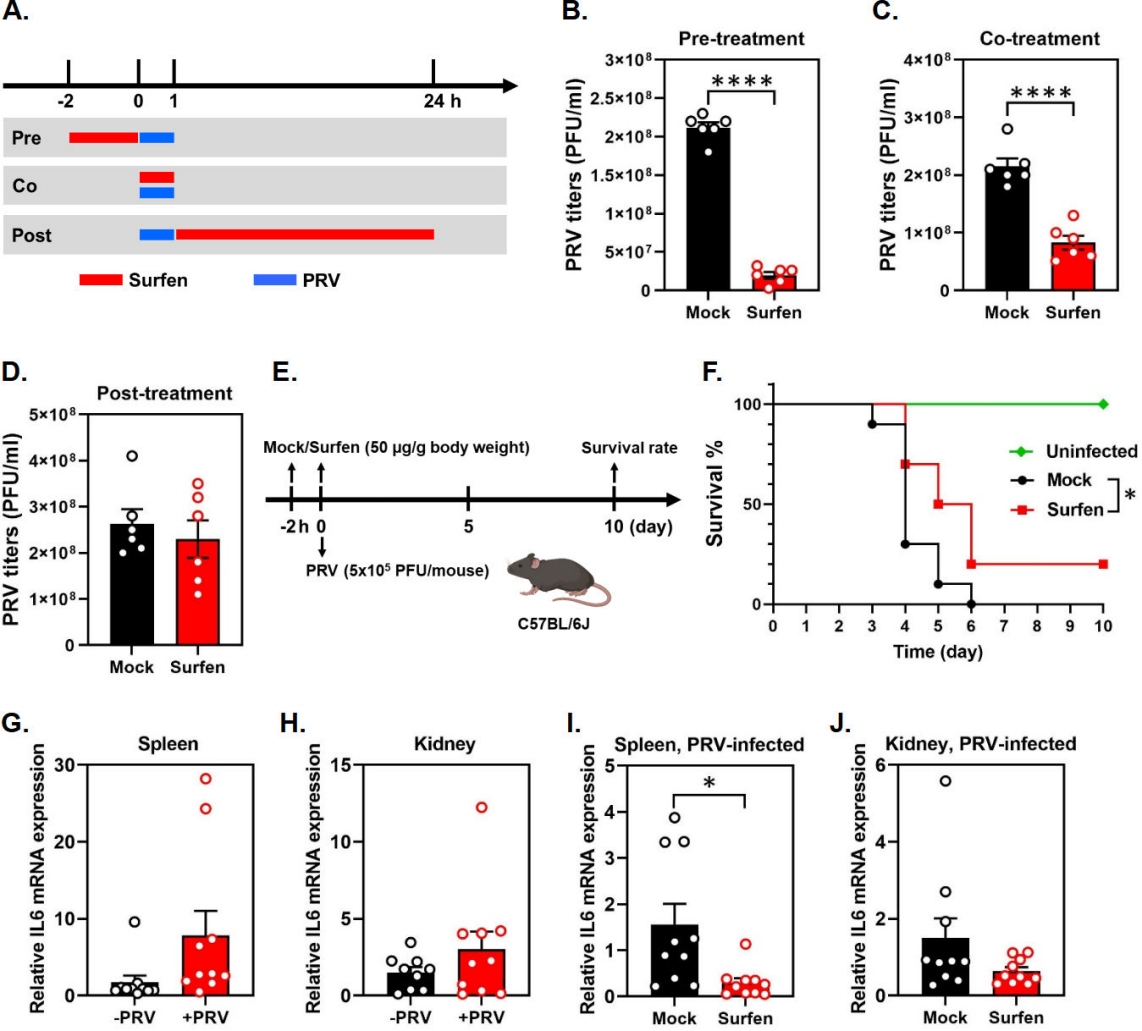
Pre-treatment of surfen inhibits PRV infection *in vitro* and *in vivo*. (**A**) The schematic diagram of the drug time-of-addition experiments in PK15 cells. (**B–D**) Surfen (10 µM) was pre-treated 2 h before PRV infection (**B**), co-treated during PRV infection (**C**), or treated post-PRV infection (**D**). The cells and supernatants were harvested at 24 hpi, and viral titers were determined by plaque assay. Values represent the means ± SEM of two independent experiments. Significance was determined by a two-tailed, unpaired *t*-test (****P < 0.0001). (**E**) The schematic diagram of surfen antiviral experiments in mice. (**F**) Mice were mock injected, or injected with surfen intraperitoneally, and then infected with PRV at a lethal dose (5 × 10^5^ PFU/mouse) in the presence of surfen. The mice death was monitored every day until 10 days post-infection. The survival curves were then analyzed using the Log-rank (Mantel-Cox) test and Gehan-Breslow-Wilcoxon test (*P < 0.05, *n* = 3 mice for uninfected group, and *n* = 10 mice for mock and surfen groups, respectively, from three independent experiments). (**G–J**) Mice were mock treated or intraperitoneally injected with surfen and then uninfected or infected with PRV in the presence or absence of surfen. The mRNA expression levels of IL6 in the spleen and kidney were determined by RT-qPCR using the primers specific for mouse IL6. (**G and H**) Relative IL6 mRNA expression was shown as the normalization of IL6 mRNA level in the spleen (**G**) and kidney (**H**) of PRV-infected (PRV+) mice to that of PRV-uninfected (PRV-) mice. (**I and J**) Relative IL6 mRNA expression was displayed as the normalization of IL6 mRNA level in the spleen (**I**) and kidney (**J**) of surfen-injected PRV-infected mice to that of mock-treated PRV-infected mice. Values represent the means ± SEM of two independent experiments (*n* = 10 mice for each group). Significance was determined by a two-tailed, unpaired *t*-test (*P < 0.05).

Having systemically determined the inhibitory effects of surfen on PRV gene expression, DNA synthesis, and virus production, we next sought to detect the specific stage at which surfen inhibits PRV. To test this, we performed the drug time-of-addition experiments where surfen was pre-treated before PRV infection, co-treated during PRV infection, or treated post-PRV infection (Fig. 3A). We observed that surfen could inhibit PRV at the maximum when it was pre-treated in PK15 cells (Fig. 3B) compared to co-treatment (Fig. 3C) and post-treatment (Fig. 3D). It is worth noting that surfen failed to display any antiviral activity when added post-virus entry (Fig. 3D). Taken together, these findings suggest that surfen inhibits PRV infection at the virus entry stage.

Having demonstrated an inhibitory effect of surfen on PRV infection *in vitro*, we next sought to determine whether surfen also inhibited PRV infection *in vivo*. To test this, we first determined whether surfen induced toxicity in mice through histopathological examination. Staining of tissue sections by hematoxylin and eosin revealed that surfen did not cause any histopathological alterations in the heart, liver, spleen, lung, and kidney at doses of 25 and 50 µg/g body weight (Fig. S1), demonstrating that surfen does not induce toxicity in mice under these concentrations. Because PRV infection of mice causes severe neurological signs, ultimately leading to death, we determined whether surfen treatment would elevate the survival rate in mice after lethal PRV challenge (Fig. 3E). As expected, all mice died within 6 days post-PRV infection in the mock group (Fig. 3F). In contrast, surfen treatment significantly elevated mice survival rate, with 20% of mice surviving by the end of the experiments (Fig. 3F). However, surfen failed to show an inhibitory effect on PRV viral loads in all the organs tested (heart, liver, spleen, lung, kidney, and brain) (Fig. S2).

To determine whether surfen promoted mice survival by affecting PRV-induced inflammation, we detected the mRNA expression levels of well-established proinflammatory cytokines interleukin 6 (IL6) and IL18 by RT-qPCR in the representative organs (spleen and kidney). We observed that PRV infection induced the expression of IL6 in spleen and kidney (Fig. 3G and H); however, surfen showed an inhibitory effect on PRV-induced IL6 expression in these organs (Fig. 3I and J), suggesting that surfen might control PRV-induced inflammation to promote mice survival. In contrast, PRV infection failed to induce IL18 expression (Fig. S3A and B), and surfen had no impact on IL18 expression during PRV infection (Fig. S3C and D). Collectively, these findings demonstrate that surfen only displays modest antiviral activity against PRV in mice.

### Surfen inhibits PRV attachment to target cells

**Fig 4:**
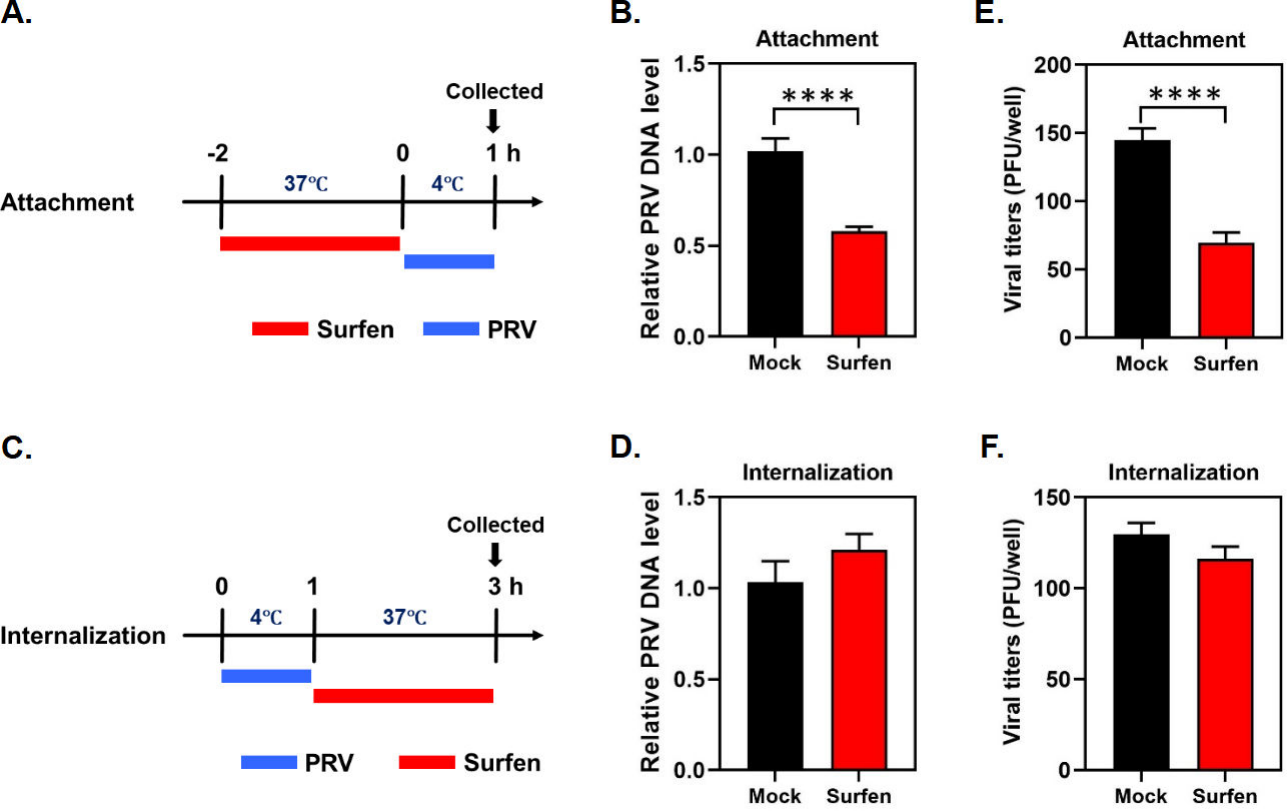
Surfen inhibits PRV attachment to target cells. (**A**) The schematic diagram of virus attachment experiments. (**B**) PK15 cells were mock treated or treated with 10 µM surfen at 37°C for 2 h, and then infected with PRV at 4°C for 1 h. DNA was extracted from the cells, and viral gB DNA copies were measured by qPCR to assess the relative PRV DNA level. (**C**) The schematic diagram of virus internalization experiments. (**D**) PK15 cells were infected with PRV at 4°C for 1 h, then mock treated, or treated with 10 µM surfen at 37°C for 2 h. DNA was extracted from the cells, and viral gB DNA copies were measured by qPCR to assess the relative PRV DNA level. (**E**) PK15 cells were prechilled at 4°C for 1 h and then infected with PRV in the presence of 10 µM surfen at 4°C for another 80 min. The cells were washed with cold PBS, and viral titers were determined by plaque assay. (**F**) PK15 cells were prechilled at 4°C for 1 h and then incubated with PRV at 4°C for another 2 h to allow virus attachment. Then, 10 µM surfen was added and incubated at 37°C for 10 min. The cells were washed with acidic buffer (pH 3) to inactivate non-penetrated virus, and viral titers were determined by plaque assay. Values represent the means ± SEM of two to three independent experiments. Significance was determined by a two-tailed, unpaired *t*-test (****P < 0.0001).

The above findings demonstrate that surfen pre-treatment before virus infection results in PRV inhibition, suggesting that surfen exerts an antiviral effect at the viral entry stage. Therefore, we determined the effect of surfen on PRV attachment and internalization by qPCR. PK15 cells were treated with surfen prior to PRV infection (Fig. 4A). Then, the virus attached to cells was detected by qPCR. We found that surfen pre-treatment resulted in a significant approximately 50% reduction in virus attachment compared to the mock group (Fig. 4B). We next infected PK15 cells with PRV to allow virus attachment to cells first and then added surfen to assess whether virus internalization was affected by qPCR (Fig. 4C). However, we observed that PRV was internalized into surfen-treated cells at a level comparable to that of mock-treated cells (Fig. 4D). To further validate whether surfen influenced PRV binding to cell surface or whether it impaired PRV penetration, we performed virus binding and penetration assays and then detected viral titers by plaque assay, following the previously reported protocols (34). We found that surfen inhibited PRV binding to the cell surface (Fig. 4E) rather than affecting PRV penetration (Fig. 4F). Taken together, these findings demonstrate that surfen inhibits PRV attachment rather than internalization.

### Inhibition of PRV attachment by surfen is dependent on HS

**Fig 5:**
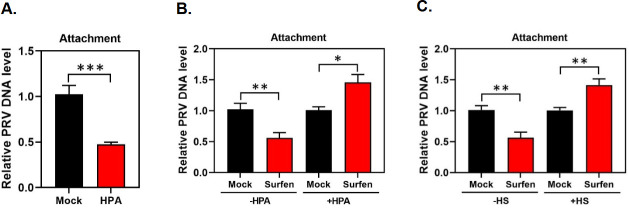
Enzymatic removal of HS chains by heparinase or addition of exogenous HS reverses PRV inhibition by surfen. (**A**) PK15 cells were mock treated or treated with heparinase at 10 units for 2 h and then infected with PRV at 4°C for 1 h. DNA was extracted from the cells, and viral gB DNA copies were measured by qPCR to assess the relative PRV DNA level. (**B**) PK15 cells were mock treated or treated with heparinase at 10 units for 2 h, followed by mock or 10 µM surfen treatment for 2 h, and then infected with PRV at 4°C for 1 h. DNA was extracted and quantified exactly as described for (**A**). (**C**) PK15 cells were mock treated or treated with 10 µM surfen in the absence or presence of porcine HS for 2 h and then infected with PRV at 4°C for 1 h. DNA was extracted and quantified exactly as described for (**A**). Values represent the means ± SEM of two independent experiments. Significance was determined by a two-tailed, unpaired *t*-test (*P < 0.05, **P < 0.01, ***P < 0.001).

Since surfen has a binding ability to HS and can inhibit various HS-mediated biological processes (31, 32), we hypothesized that surfen inhibited PRV attachment by directly blocking virions binding to HS on target cells. To test this, we determined whether removal of HS chains on the surface of PK15 cells by heparinase (HPA) resulted in a loss in the ability of surfen to inhibit PRV. It has been previously demonstrated that HPA treatment led to a significant reduction in PRV attachment to target cells (6, 9). Therefore, we first treated PK15 cells with HPA, followed by PRV infection, to validate the effect of HPA on PRV attachment. In agreement with previous findings (6, 9), we also observed that PRV attachment was significantly decreased post-HPA treatment (Fig. 5A). Subsequently, we determined the inhibitory effect of surfen on PRV attachment after the removal of HS chains by HPA. Consistent with our above findings (Fig. 4B), surfen has an inhibitory effect on PRV attachment in the absence of HPA (Fig. 5B). However, interestingly, we found that surfen lost its ability to inhibit PRV attachment after HPA treatment (Fig. 5B). These findings indicate that surfen inhibits PRV attachment by disrupting virions binding to HS on the surface of PK15 cells.

To further validate the above findings, we determined whether addition of exogenous HS to neutralize surfen reversed the effect of surfen on PRV inhibition. To test this, porcine HS and surfen were mixed first and then incubated to allow their binding before being added to PK15 cells. Following infection, PRV attachment was quantified by qPCR. Consistent with our above findings (Fig. 4B and 5B), surfen has an inhibitory effect on PRV attachment in the absence of HS. In contrast, neuralization of surfen by addition of exogenous HS resulted in a loss in the ability of surfen to inhibit PRV (Fig. 5C). Cumulatively, these findings demonstrate that surfen inhibits PRV attachment by blocking virions binding to HS on target cells.

### Surfen fails to inhibit the attachment of the PRV gC-deficient virus

**Fig 6:**
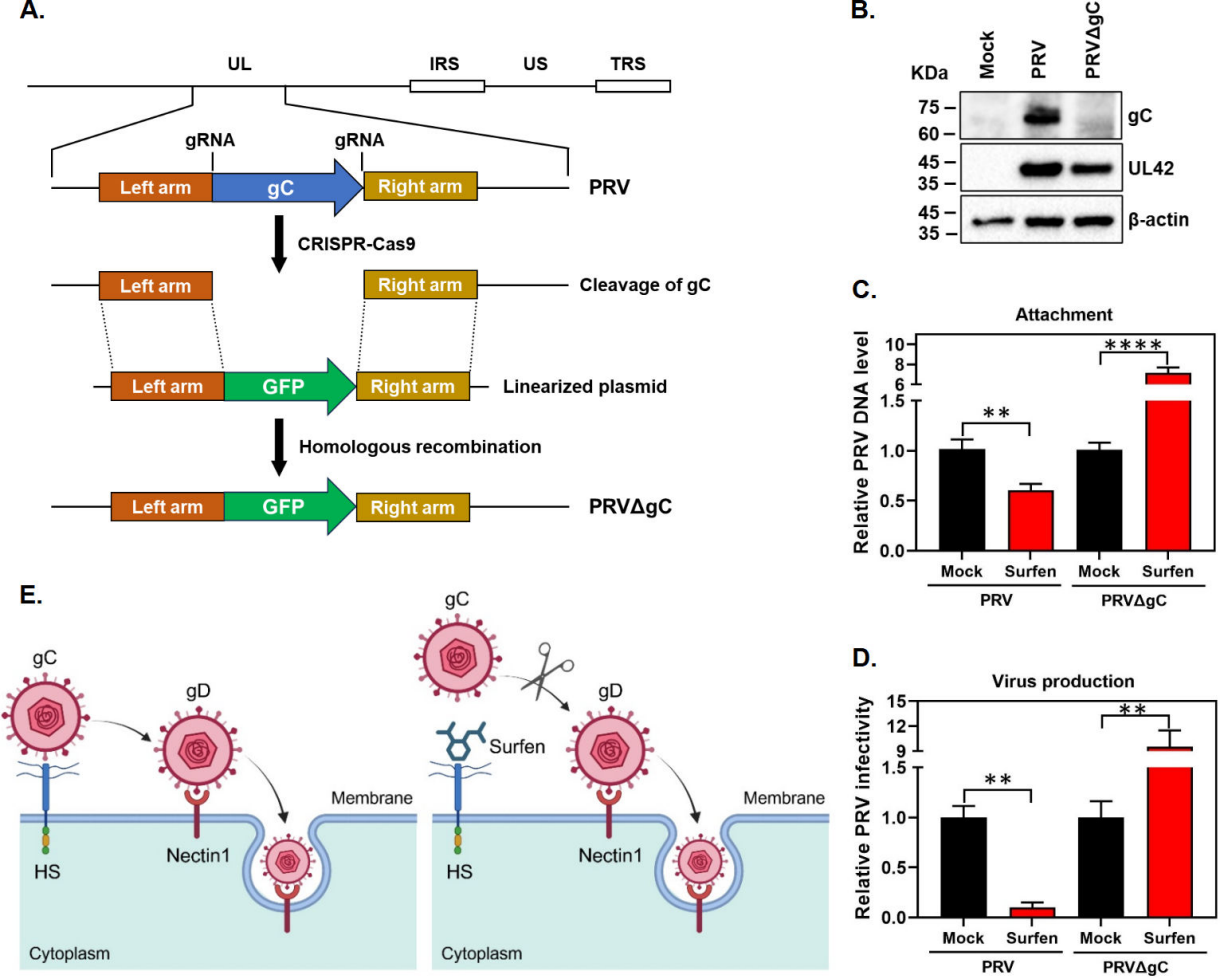
Surfen fails to inhibit the attachment of the PRV gC-deficient virus. (**A**) Schematic diagram for constructing the PRV gC-deficient virus (PRVΔgC). (**B**) PK15 cells were mock infected or infected with PRV or the PRVΔgC virus for 24 h, then the protein lysates were harvested, and the expression levels of PRV gC and UL42 proteins were determined by Western blots. β-Actin was included as a loading control. (**C**) PK15 cells were mock treated or treated with 10 µM surfen at 37°C for 2 h and then infected with PRV or PRVΔgC at 4°C for 1 h. DNA was extracted from the cells, and viral gB DNA copies were measured by qPCR to assess the relative PRV DNA level. (**D**) PK15 cells were mock treated or treated with surfen at 10 µM for 2 h, infected with PRV or PRVΔgC in the presence of surfen for 1 h, and then incubated in the continued presence of surfen for 24 h. The cells and supernatants were harvested, and then viral titers were measured by plaque assay. Values represent the means ± SEM of two independent experiments. Significance was determined by a two-tailed, unpaired *t*-test (**P < 0.01, ****P < 0.0001). (**E**) A proposed working model for inhibition of PRV attachment by surfen. Left panel: In the absence of surfen, PRV attaches to target cells by binding its viral glycoprotein gC to HS, then the viral glycoprotein gD interacts with one of the many cellular receptors, including nectin 1, to mediate membrane fusion and viral entry. Right panel: In the presence of surfen, PRV gC fails to bind HS on target cells due to surfen interaction with HS, thereby resulting in a block in virus attachment. The working model was created with Biorender.com.

PRV gC has been proven to be the major viral glycoprotein responsible for binding to HS on target cells (6–10), and the PRV gC-deficient virus utilizes an HS-independent pathway to enter cells (6, 7). Thus, we speculated that surfen would lose its ability to inhibit PRV in the absence of gC. To test this, we determined the effect of surfen on the attachment and infectious virus production upon PRV gC-deficient virus infection. To do this, we first constructed the PRV gC-deficient virus by replacing PRV gC with the GFP expression cassette using the CRISPR-Cas9 and homologous recombination (Fig. 6A). Through western blot assay using an antibody directed against PRV gC, we validated that the expression of PRV gC protein has already disappeared following infection with the PRV gC-deficient virus (Fig. 6B). Subsequently, we measured the effect of surfen on the attachment of this gC deletion mutant virus by qPCR. Not surprisingly, we observed that surfen did not display any inhibitory effect on the attachment of PRV with the deletion of gC (Fig. 6C). Furthermore, surfen did not inhibit the production of PRV gC-deficient virus particles (Fig. 6D). As controls, surfen indeed showed an inhibitory effect on PRV attachment and infectious virus production (Fig. 6C and D), in agreement with our above findings (Fig. 1C and 4B). Cumulatively, these findings reveal a working model where, in the presence of surfen, the HS on target cells was occupied so that PRV gC has no place to bind, leading to reduced virus attachment and decreased virus production (Fig. 6E, right panel). In contrast, in the absence of surfen, PRV gC binds to HS on target cells, enabling the virus to complete the attachment process smoothly (Fig. 6E, left panel).

## DISCUSSION

PRV infection results in severe respiratory, reproductive, and neurological disorders in pigs and many other animals. PRV can also infect humans, but relevant reports are very rare, and its pathogenesis in humans is still unclear. PRV is not merely a serious concern in veterinary medicine, but also a potential threat to public health. Therefore, the development of effective antiviral agents against PRV is important for preventing its infection and spread. In the work presented here, we identified surfen as a novel and potent antiviral agent against PRV infection in multiple permissive cells *in vitro* and in mice *in vivo*. We demonstrated that surfen prevented the attachment of PRV virions to target cells, which is dependent on HS. However, surfen did not interfere with the attachment of PRV gC-deficient virus, which enters cells in an HS-independent manner. Cumulatively, these findings demonstrate that surfen inhibits PRV infection by preventing the binding of PRV gC to HS on target cells.

Numerous viruses utilize HS as an attachment receptor for binding to the surface of target cells. This makes HS an ideal target for the development of antiviral drugs. Therefore, small-molecule inhibitors that block the interaction of HS and virions could prove useful as therapeutic candidates. We examined the antiviral effect of surfen on PRV due to its binding ability to HS and the findings that PRV attaches to target cells by binding to cell surface HS. Here, we demonstrated that surfen is a good drug candidate against PRV infection. Consistent with our findings that surfen inhibits PRV infection, an early study also reported that surfen repressed HS-dependent infection with HSV-1, an important human alphaherpesvirus (31). However, which HSV-1 viral glycoproteins surfen competes with for HS binding, and whether surfen could exert an inhibitory effect on HSV-1 infection *in vivo*, remain elusive. In addition, surfen has been demonstrated to inhibit the enhancement of HIV-1 infection by semen-derived amyloid fibrils through interfering with their binding to HS on target cells (32). However, it is noteworthy that although surfen can block HS-dependent activities, the research on its antiviral activity against virus infection remains very limited. Therefore, the antiviral activity of surfen against viruses other than PRV, HSV-1, and HIV-1 warrants significant and extensive investigations.

Surfen is a member of the family of aminoquinoline drugs that was first developed as an excipient for the production of depot insulin and was subsequently demonstrated as a potent HS antagonist (29, 31). In addition to surfen, several antimalarial agents, including chloroquine and amodiaquine, are important members in the family of aminoquinoline drugs. Apart from the potent antimalarial activities, chloroquine and amodiaquine have been shown to inhibit the infections of multiple viruses, including severe acute respiratory syndrome coronavirus 2, influenza A virus, dengue virus, Zika virus, and Ebola virus (35–40). It is worth noting that, like surfen, chloroquine and amodiaquine are also the 4-aminoquinoline class of compounds, implicating that they might share conserved functions during virus infection. However, to date, whether the other aminoquinoline drugs, such as chloroquine and amodiaquine, also displayed antiviral activities against PRV infection has not been determined. Therefore, it will be very interesting to determine whether other aminoquinoline drugs, especially the antimalarial agents, could effectively inhibit PRV infection *in vitro* and *in vivo*.

In the work described here, we present the evidence to demonstrate that blocking the binding of PRV virions to target cells is an effective strategy to develop candidate antiviral drugs against PRV infection. In fact, researchers made great efforts to identify specific inhibitors that disrupt the binding of PRV virions to target cells in recent years. A representative example is that the 25 kDa linear polyethylenimine has been proven to exert a potent antiviral activity against PRV infection by interfering with virus adsorption via electrostatic interaction (41). In another study from the same group, it was found that *Rehmannia glutinosa* polysaccharide exerts a good antiviral activity against PRV infection by preventing virus attachment (42), although the specific mechanism by which this polysaccharide interferes with virus absorption remains unclear. Moreover, *lysimachia christinae* polysaccharide has also been demonstrated to impair PRV infection by downregulating attachment, probably through direct killing of PRV viral particles (43). Interestingly, a high-throughput screen of a natural product chemical library identified that salvianolic acid A exhibited an inhibitory effect on RPV infection by directly inactivating viral particles to block virus attachment (44). Taken together, these findings demonstrate that blocking the attachment of PRV virions to target cells is a feasible strategy to develop efficient antiviral drugs against PRV infection. Thus, it is important to promote the already proven efficient antiviral drugs targeting PRV attachment to the clinical trials in time.

A surprising and interesting finding in this work is that surfen induces enhancement of PRV attachment when the primary binding sites (HS or gC) are blocked. When the HS on the cell surface was depleted with HPA, PRV attachment after surfen treatment was significantly elevated (Fig. 5B). Furthermore, when the PRV *gC* gene was deleted from the virus, the attachment and infection of PRV gC-deficient virus were strikingly increased following surfen treatment (Fig. 6C and D). The specific mechanism underlying this remains unclear, but we speculated that surfen might activate an alternative route to promote PRV entry when the primary PRV entry pathway mediated by HS-gC interaction is blocked. This potential alternative route is unknown for now and is worth further investigation in the future.

In the work presented here, surfen was demonstrated to display a good inhibitory effect on PRV attachment and viral particle production in cell culture *in vitro* (Fig. 1 to 4); however, it did not reduce viral loads in the organs of PRV-infected mice *in vivo* (Fig. S2). The specific mechanisms underlying this discrepancy remain unclear, but we speculated that the possible reasons may include a couple of aspects. First, the *in vivo* environment is far more complex than the simplified *in vitro* cell culture conditions. PRV may employ multiple non-HS-dependent pathways to enter target cells in mice, which makes surfen, when distributed throughout the organs, less efficient in blocking all potential virus binding sites compared to its action in cell culture. Second, the number of vulnerable target cells in PRV-infected mice may be much higher than the limited number of cells in cell culture, which might overwhelm surfen’s inhibitory effect, neutralizing the benefit of reducing initial attachment. Thus, it is likely that the ability of surfen to prevent PRV entry is masked *in vivo*.

On the other hand, we observed that surfen reduced IL6 mRNA expression in PRV-infected spleen and kidney (Fig. 3I and J), inhibiting PRV-induced inflammation in mice, while it failed to repress viral loads in PRV-infected mice (Fig. S2). These results indicate that the primary effect of surfen seems to be anti-inflammatory rather than directly antiviral in the mouse model of PRV infection. While direct evidence that surfen inhibits virus-induced inflammation has not been reported, it has been recently demonstrated that surfen reduced the mRNA expression levels of several proinflammatory cytokines, including IL6, in bone marrow-derived macrophages and repressed inflammation in the mouse model of multiple sclerosis (45). Moreover, surfen produced sustained analgesic activity when delivered intrathecally in mouse models of acute and chronic inflammatory pain (46). Thus, surfen appears to act as a broad anti-inflammatory agent that modulates inflammatory response of the host immune system. The specific mechanisms by which surfen inhibits inflammation remain unclear, but it is plausible that it (i) directly reduces the production of proinflammatory cytokines and chemokines, including IL6, C-C motif chemokine ligand 2 (CCL2), CCL4, and CCL5, to inhibit the overall inflammatory responses (45); (ii) directly binds to glycosaminoglycans to prevent forming the chemokine gradients that recruit immune cells to the sites of inflammation; and (iii) blocks receptor binding of the anaphylatoxin complement component 5a (C5a), a potent mediator of acute inflammation, to reduce inflammatory responses (47). Taken together, these findings suggest that surfen may provide partial protection against PRV infection in mice through its anti-inflammatory activity.

In summary, the findings described here reveal surfen as a novel and potent antiviral agent against PRV infection. Surfen inhibits PRV attachment by preventing the binding of PRV gC to HS on target cells. This work reinforces the concept that targeted disruption of PRV gC-HS interaction is an effective strategy to develop antiviral drugs to defend PRV infection.

## MATERIALS AND METHODS

### Cell culture

BHK21, PK15, 293T, and Neuro-2a cells were grown and maintained in high-glucose Dulbecco’s Modified Eagle Medium (DMEM; Seven Biotech, SC102-02) supplemented with 10% fetal bovine serum (FBS; EXCell, FSP500) and 1× penicillin-streptomycin solution (Beyotime, C0222) at 37°C with 5% CO_2_.

### Generation of the PRV gC-deficient virus

The PRV gC-deficient (PRVΔgC) virus was generated by replacing PRV gC with green fluorescent protein (GFP) on the backbone of the PRV wild-type virus (the HLJ8 strain, GenBank no. KT824771.1) (48), using the small guide RNAs (gRNAs) and primers as previously described (49). Briefly, the gRNAs used for cleaving PRV gC were synthesized and annealed and then cloned into the pX330 vector to generate the pX330-gC-gRNA plasmid for the expression of PRV gC gRNAs and Cas9. The homologous left and right arms were amplified from the PRV genomic DNA, and the recombinant transfer plasmid carrying the GFP expression cassette flanked by the homologous arms was generated by fusion PCR. To generate the PRVΔgC virus, BHK21 cells were co-transfected with 4 µg of PRV genomic DNA, 0.5 µg of pX330-gC-gRNA plasmid, and 0.5 µg of linearized recombinant transfer plasmid using PEI transfection reagent. The recombinant virus expressing GFP was harvested when 50–60% of cytopathic effect was observed. The virus was then purified by five rounds of plaque assays. The resulting PRVΔgC virus was validated by DNA sequencing and western blots to ensure the deletion of PRV gC.

### Cell viability assay

PK15, 293T, and Neuro-2a cells were seeded in 96-well plates, mock treated, or treated with surfen (MedChemExpress, HY-122704A, 1.25, 2.5, 5, 10, and 20 µM for PK15 cells, 10 µM for 293T cells, and 10 µM for Neuro-2a cells), and then incubated at 37°C for 24 h. Cell viability was determined using a WST-8 based Cell Counting Kit-8 (CCK-8; Biosharp, BS350B), following the manufacturer’s instructions. Absorbance was measured at 450 nm using a Varioskan Flash spectral scanning multimode reader (Thermo Fisher Scientific).

### Virus infection

One day prior to infection, PK15, 293T, and Neuro-2a cells were seeded at 3 × 10^5^ cells per well in 12-well plates. The cells were mock treated or treated with surfen at the indicated concentrations for 2 h, then infected with PRV at a multiplicity of infection (MOI) of 0.1 or 1 for 1 h in the presence of surfen, and then incubated in the continued presence of surfen. At the indicated time points post-infection: (i) the cells and supernatants were collected and subjected to two cycles of freezes and thaws prior to the detection of viral titers by plaque assay; (ii) the cells were harvested and subjected to RNA extraction for gene expression analyses by reverse transcription-quantitative PCR (RT-qPCR); and (iii) the cells were collected and lysed, and the protein lysates were prepared for protein expression analyses by Western blots.

### Plaque assay

One day prior to infection, PK15 cells were seeded at 3 × 10^5^ cells per well in 12-well plates. The viral samples were serially 10-fold diluted in serum-free DMEM, added to the plates in triplicate, and incubated for 1 h. Then, the cells were overlaid with a 1:1 mixture of 2.5% methyl cellulose (Sangon Biotech, A600616-0250) and 2× DMEM (MesGen Biotech, MDC1102) supplemented with 2% FBS and 1× penicillin-streptomycin solution and incubated for three days. The cells were fixed, and the plaques were stained with a fixation and crystal violet staining solution (2% formaldehyde, 20% ethanol, 0.01% crystal violet, and 6.75% sodium chloride). The number of plaques was counted and viral titers were then calculated.

### RT-qPCR and qPCR

For quantification of viral gene expression, total RNA was extracted using the UE Multisource Total RNA Minprep Kit (US Everbright, UE-MN-MS-RNA-250) according to the manufacturer’s protocols. First-strand cDNA synthesis was performed with 1 μg of total RNA using the EasyScript All-in-One First-Strand cDNA Synthesis SuperMix for qPCR (One-Step gDNA Removal) (TransGen Biotech, AE341-02), following the manufacturer’s instructions. All qPCRs were conducted in triplicate using the PerfectStart Green qPCR SuperMix (TransGen Biotech, AQ601-02-V2) using the primers specific for PRV *IE180* (Forward: 5′-TCGTGCGCCTCATCTACA-3′; Reverse: 5′-GCTGGCAGAACTGGTTGAA-3′), PRV *UL42* (Forward: 5′-CACGCCTTCCTCGTCTTC-3′; Reverse: 5′-ACACGAACGTGCTGAACT-3′), PRV *gB* (Forward: 5′-GACAACGAGCTCCTCATCTC-3′; Reverse: 5′-ACGTAGCTGTAGTCCTCGTA-3′), and pig glyceraldehyde-3-phosphate dehydrogenase (GAPDH, Forward: 5′-ACCTCCACTACATGGTCTAC-3′; Reverse: 5′-GATGGCCTTTCCATTGATGA-3′). GAPDH was used as an internal control, and the relative expression levels of viral genes to GAPDH were determined by the comparative *C_T_* method (50).

For detection of viral DNA synthesis, DNA was extracted using the TIANamp Genomic DNA kit (TIANGEN BIOTECH, DP304-03) according to the manufacturer’s protocols, and then, qPCR was performed as described above to quantify the viral genomic DNA using the primers specific for PRV *gB* (Forward: 5′-GACAACGAGCTCCTCATCTC-3′; Reverse: 5′-ACGTAGCTGTAGTCCTCGTA-3′). Pig GAPDH DNA was detected as an internal control using the primers (Forward: 5′-GACCTCAGCTCTTAGCAAAC-3′; Reverse: 5′-CATCAGAGAACCATCCAGAAA-3′). The PRV DNA level was normalized relative to GAPDH DNA.

### Western blots

The cells were washed with ice-cold phosphate-buffered saline (PBS; KeyGEN BioTECH, KGL2206-500), lysed using Cell Lysis Buffer for Western blot and IP (Beyotime, P0013) supplemented with 1 mM phenylmethanesulfonyl fluoride (PMSF; Beyotime, ST506), and then clarified by centrifugation. Protein lysates were quantified using the Enhanced BCA Protein Assay Kit (Beyotime, P0010). The protein samples were mixed with 6 × Protein Loading Buffer (TransGen Biotech, DL101) and denatured at 100°C for 10 min. The protein samples were separated on 10% SDS-polyacrylamide gels (BAIHE, PG112, PG113) and transferred onto the Immun-Blot PVDF membranes (Bio-Rad, #1620177). The membranes were blocked with 5% non-fat dry milk in Tris-buffered saline with 0.1% Tween-20 (TBST) for 1 h at room temperature, followed by overnight incubation at 4°C with primary antibodies against UL42 (1:5,000, clone 7C11) (51), gB (1:5,000, clone 1E7) (52), gC (1:5,000; DAYAO SHENGWU, Ab0104), and β-actin (1:50,000; ABclonal, AC026). After five washes with TBST, the membranes were incubated with horseradish peroxidase (HRP)-conjugated AffiniPure Goat Anti-Mouse IgG (H+L) (1:10,000; BOSTER, BA1051) for 1 h at room temperature. After the final three washes with TBST, the protein bands were developed by the Molecular Imager ChemiDoc XRS+ Imaging System (Bio-Rad) using the Clarity Western ECL Substrate (Bio-Rad, #170-5061).

### Mice experiments

Seven- to 8-week-old C57BL/6J mice were purchased from Beijing Vital River Laboratory Animal Technology Co., Ltd. The animal protocol was approved by the Institutional Animal Care and Use Committee at the Sichuan Agricultural University. To determine the toxicity of surfen *in vivo*, mice were mock treated or intraperitoneally injected with surfen at 25 or 50 µg/g body weight. The organs (heart, liver, spleen, lung, and kidney) were harvested at 3 days post-infection, and then, the histopathological changes were examined by hematoxylin and eosin staining. To determine whether surfen inhibited PRV *in vivo*, mice were mock treated or intraperitoneally injected with surfen at 50 µg/g body weight for 2 h, and then infected with PRV intraperitoneally at 5 × 10^5^ PFU/mouse in the presence of surfen. Mice were monitored for death every day until 10 days post-infection. Meanwhile, the organs (heart, liver, spleen, lung, kidney, and brain) were harvested, and the viral loads were detected by qPCR using the primers specific for PRV *gB* (Forward: 5′-GACAACGAGCTCCTCATCTC-3′; Reverse: 5′-ACGTAGCTGTAGTCCTCGTA-3′). The mRNA expression levels of proinflammatory cytokines IL6 and IL18 in the spleen and kidney were determined by RT-qPCR, using the primers specific for mouse *IL6* (Forward: 5′-GATAAGCTGGAGTCACAGAAGG-3′; Reverse: 5′-TTTGCCGAGTAGATCTCAAAGT-3′), mouse *IL18* (Forward: 5′-TCTACCCTCTCCTGTAAGAACA-3′; Reverse: 5′-CTGGAACACGTTTCTGAAAGAAT-3′), and mouse GAPDH (Forward: 5′-CATGGCCTTCCGTGTTCCTA-3′; Reverse: 5′-CCTGCTTCACCACCTTCTTGAT-3′). GAPDH was used as an internal control, and the relative mRNA expression levels of mouse *IL6* and *IL18* genes to GAPDH were determined by the comparative *C_T_* method (50).

### Virus attachment and internalization assay

One day prior to infection, PK15 cells were seeded at 3 × 10^5^ cells per well in 12-well plates. For the attachment assay, the cells were mock treated or treated with 10 µM surfen at 37°C for 2 h and then infected with PRV (MOI = 1) at 4°C for 1 h. After washing with prechilled PBS, the cells were subjected to DNA extraction. For the internalization assay, the cells were infected with PRV (MOI = 1) at 4°C for 1 h. After washing the unbound virus with PBS, the cells were mock treated or treated with 10 µM surfen at 37°C for 2 h. DNA was extracted after washing the cells with citrate buffer (40 mM sodium citrate, 10 mM KCl, 135 mM NaCl, pH = 3.0) to remove any viruses that do not enter the cells. The relative PRV DNA level was quantified as described above.

### Statistical analyses

All data were analyzed using GraphPad Prism 9 software (GraphPad, San Diego, CA). Statistical significance was determined using a two-tailed, unpaired Student *t*-test, and *P* values less than 0.05 were considered to be statistically significant. *, *P* < 0.05; **, *P* < 0.01; ***, *P* < 0.001; and ****, *P* < 0.0001.

## Data Availability

All data are contained within the manuscript.

## SUPPLEMENTAL MATERIAL

Supplemental figures spectrum.03223-25-s0001.pdf Figures S1 to S3.

ASM does not own the copyrights to Supplemental Material that may be linked to, or accessed through, an article. The authors have granted ASM a non-exclusive, world-wide license to publish the Supplemental Material files. Please contact the corresponding author directly for reuse.
