# Supplementary material for: Aminoquinoline surfen inhibits pseudorabies virus attachment by preventing the binding of glycoprotein C to heparan sulfate
Source: Microbiol Spectr. 2026 Mar 5;14(4):e03223-25. doi: 10.1128/spectrum.03223-25 (PMC13055252; doi:10.1128/spectrum.03223-25)
Supplement: Supplemental figures — Figures S1 to S3. [file spectrum.03223-25-s0001.pdf]

Supplemental Figures

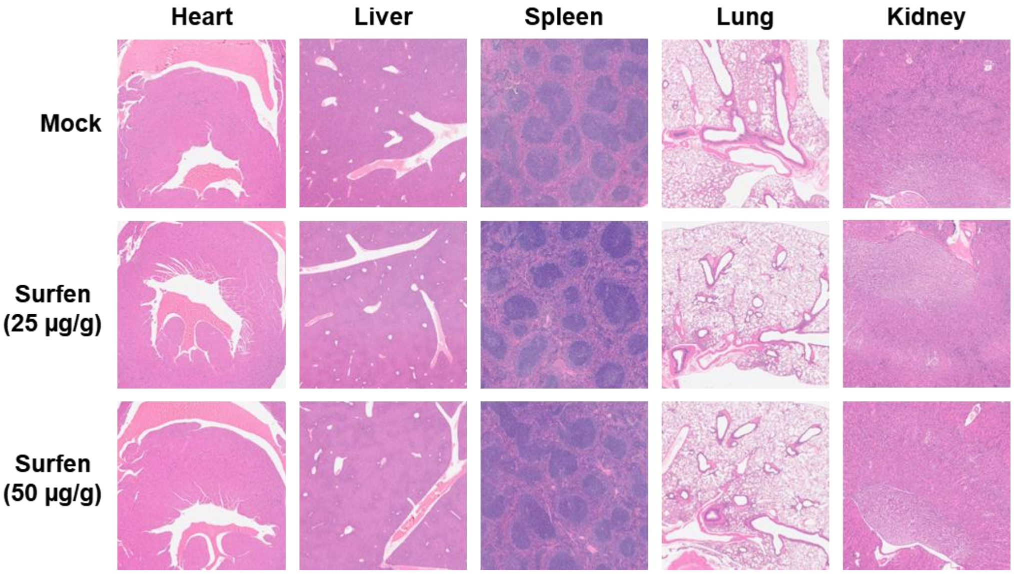

Figure S1. Surfen does not induce toxicity in mice. Mice were mock treated or intraperitoneally injected with surfen at 25 or 50 µg/g body weight, then the different organs (heart, liver, spleen, lung, and kidney) were harvested, and histopathology was examined by hematoxylin and eosin staining (3 mice/group). The representative images are shown.

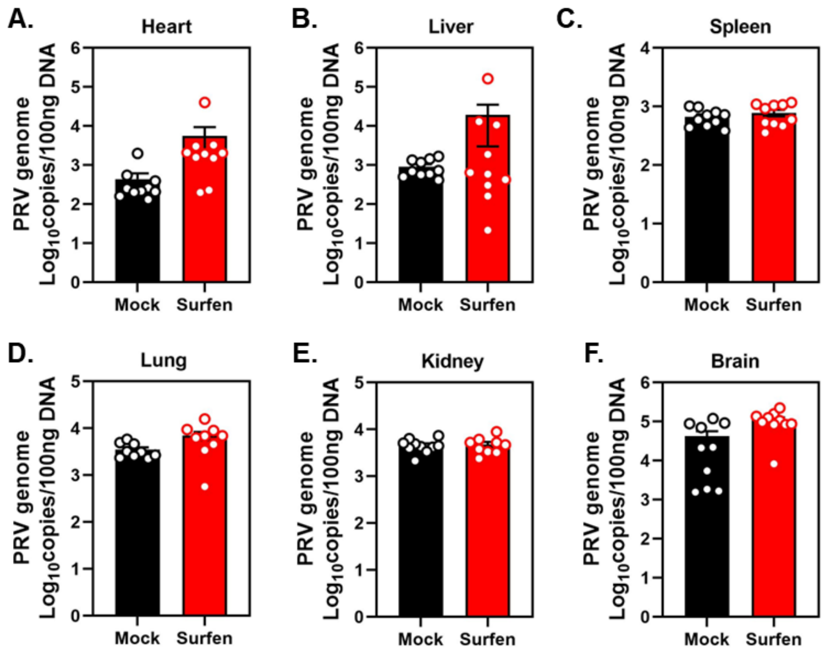

Figure S2. Surfen does not affect PRV viral loads in mice. (A-F) Mice were mock treated or intraperitoneally injected with surfen, then infected with PRV in the presence of surfen. Heart (A), liver (B), spleen (C), lung (D), kidney (E), and brain (F) were harvested, and the viral loads were detected by qPCR using the primers specific for PRV *gB* gene. Values represent the means ± the SEM of three independent experiments (n=10 mice for each group).

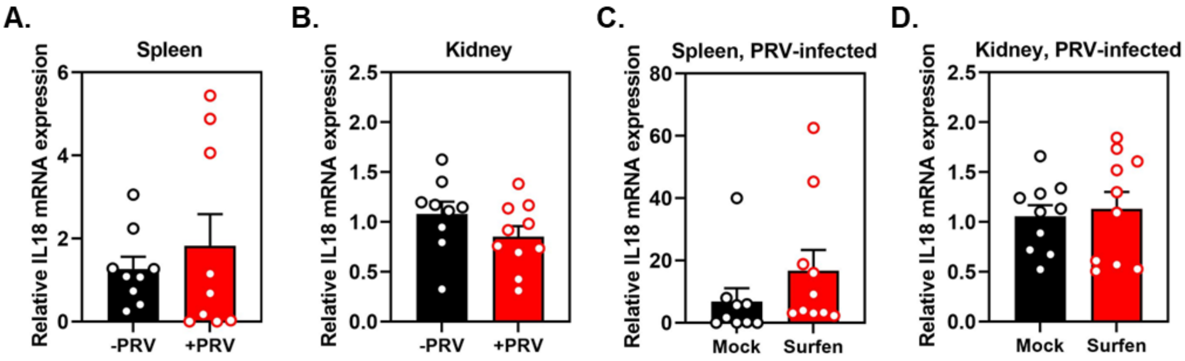

Figure S3. Surfen does not affect the expression of IL18. (A-D) Mice were mock treated or intraperitoneally injected with surfen, then uninfected or infected with PRV in the presence or absence of surfen. The mRNA expression levels of IL18 in the spleen and kidney were determined by RT-qPCR using the primers specific for mouse *IL18* gene. Values represent the means ± the SEM of two independent experiments (n=10 mice for each group).
